# Genetic and environmental contributions to population group differences on the Raven's Progressive Matrices estimated from twins reared together and apart

**DOI:** 10.1098/rspb.2007.0461

**Published:** 2007-05-11

**Authors:** J. Philippe Rushton, Trudy Ann Bons, Philip A Vernon, Jelena Čvorović

**Affiliations:** 1 Department of Psychology, University of Western OntarioLondon, Ontario, Canada N6A 5C2, Canada; 2 Serbian Academy of Sciences11000 Belgrade, Serbia

**Keywords:** behaviour genetics, cognition, cross-cultural validity, intelligence, twins

## Abstract

We carried out two studies to test the hypothesis that genetic and environmental influences explain population group differences in general mental ability just as they do individual differences within a group. We estimated the heritability and environmentality of scores on the diagrammatic puzzles of the Raven's Coloured and/or Standard Progressive Matrices (CPM/SPM) from two independent twin samples and correlated these estimates with group differences on the same items. In Study 1, 199 pairs of 5- to 7-year-old monozygotic (MZ) and dizygotic (DZ) twins reared together provided estimates of heritability and environmentality for 36 puzzles from the CPM. These estimates correlated with the differences between the twins and 94 Serbian Roma (both *r*s=0.32; *N*s=36; *p*s<0.05). In Study 2, 152 pairs of adult MZ and DZ twins reared apart provided estimates of heritability and environmentality for 58 puzzles from the SPM. These estimates correlated with the differences among 11 diverse samples including (i) the reared-apart twins, (ii) another sample of Serbian Roma, and (iii) East Asian, White, South Asian, Coloured and Black high school and university students in South Africa. In 55 comparisons, group differences were more pronounced on the more heritable and on the more environmental items (mean *r*s=0.40 and 0.47, respectively; *N*s=58; *p*s<0.05). After controlling for measurement reliability and variance in item pass rates, the heritabilities still correlated with the group differences, although the environmentalities did not. Puzzles found relatively difficult (or easy) by the twins were those found relatively difficult (or easy) by the others (mean *r*=0.87). These results suggest that population group differences are part of the normal variation expected within a universal human cognition.

## 1. Introduction

Ever since Galton (1869) and his cousin Darwin (1871), there has been debate over whether general mental ability (GMA) is an innate, cultural universal or is specific to population, time and place. With the growth of evolutionary psychology, innate universalism has regained ground after six decades of being out of fashion (Pinker 2002). One question this paper addresses is ‘How universal are psychological theories?’ Specifically, ‘Are group differences influenced by the same transaction of genetic and ecological factors as individual differences within a group?’ It is well established that individual differences in GMA, at least within the White populations of the First World, are 50–80% heritable (Jensen 1998; Bouchard & McGue 2003). The smaller amount of data available for East Asian populations, and for the Black population of the US, yield similar values (Rushton & Jensen 2005). However, the mean differences *between* groups are often postulated to be due to specific ecological factors and specialized cognitive styles (Nell 2000; Kim *et al.* 2006). As the trend towards a more global economy continues, mean group differences in GMA are likely to become more salient, both within and across countries (Lynn 2006).

In this paper, two studies based on independent twin samples are used to calculate estimates of *heritability* (an indicator of genetic influence) and *environmentality* (an indicator of non-genetic influence) for scores on the diagrammatic puzzles that make up the Raven's Progressive Matrices. These estimates are then correlated with differences calculated between diverse groups on the same test items. Strong inference is possible (Platt 1964). (i) Genetic theory predicts a positive association between heritabilities and group differences, (ii) ecological theory predicts a positive association between environmentality and group differences, and (iii) many models predict that both genetic and environmental factors contribute independently. However, extreme culture-only theories, which emphasize non-universality, predict a zero relationship between heritability and group differences (Gould 1996; Nell 2000).

To simplify, it is assumed that monozygotic (MZ) twins share 100% of their genes, while dizygotic (DZ) twins share only 50%. When the twins are reared together, they are assumed to share environmental influences, but when reared apart, they are not. Heritabilities and environmentalities are then estimated from these twin similarities and differences (Plomin *et al.* 2001; Bouchard & McGue 2003). In Study 1, the twins were reared together (MZT/DZT). Heritability was estimated by 2×(MZT*r*−DZT*r*), i.e. doubling the difference between the MZT and DZT similarities, and two environmentalities were estimated: *shared* family effects by MZT*r*-heritability and *non-shared* family effects by *Σ*|MZT1-MZT2|, i.e. the sum of all the MZT pair differences, with the differences between the twins assumed to be due to the environment. In Study 2, the twins were reared apart (MZA/DZA). Four heritability estimates were calculated: (i) 2×(MZA*r*−DZA*r*), i.e. doubling the difference between the MZA and DZA similarities, (ii) the MZA*r* itself, (iii) 2×DZA*r*, and (iv) the average of the three. Environmentality was estimated by *Σ*|MZA1−MZA2|, the sum of all the MZA pair differences.

Jensen (1998) proposed the *method of correlated vectors* for determining whether there is an association between a column of quantified elements (such as heritabilities and environmentalities) and any parallel column of independently derived scores (such as differences between groups). Previous studies have taken vectors of heritabilities and environmentalities from twins and other family members beyond the immediate data and found, in studies of mate choice, liking and friendship, that similarity between partners was more pronounced on the more heritable items within the sets of homogeneous traits (Tesser 1993; Rushton & Bons 2005). Among anthropometric measures, for example, wrist size is more heritable than biceps size because osseous parts of the body are less susceptible to environmental modification than muscular parts. Other studies have investigated US group differences in GMA. For example, P. L. Nichols (1972) unpublished data found a correlation of *r*=0.67 (*p*<0.05) between heritabilities for 13 tests estimated from twins and the magnitude of mean White–Black differences. Jensen (1973) found an inverse relation of *r*=−0.70 (*p*<0.01) between environmentality for 16 tests estimated from siblings and the mean White–Black differences. Rushton (1989) found a correlation of *r*=0.48 (*p*<0.05) between genetic influence on 11 tests estimated from inbreeding depression in cousin marriages in Japan and White–Black differences in the US.

## 2. Material and methods

The Raven's Progressive Matrices are the most well-known and best researched of all culture-reduced tests of GMA. Two versions of the test are used. Both consist of diagrammatic puzzles, each with a missing part, which the test taker attempts to identify from several options. The *Coloured* Progressive Matrices (CPM) consist of 36 puzzles presented in colour. Since this test spreads out the scores of the bottom 20% of the general population, it is typically given to young children (Raven *et al.* 1995). The *Standard* Progressive Matrices (SPM) consists of 60 non-coloured puzzles suitable for a middle range of ability (Raven *et al.* 1998). The first 24 puzzles are the same in both the CPM and the SPM (although the CPM presents them in colour). Reliability and validity remain high across a wide variety of cultural groups, regardless of whether a timed or untimed assessment is administered. Both the CPM and the SPM are good measures of *g*, the general factor of GMA (Jensen 1998). These tests have been described as measuring ‘analogical thinking’, ‘the ability to identify relationships’ and to ‘think clearly’ (Raven *et al.* 1998).

### (a) Study 1: 199 pairs of 5- to 7-year-old Canadian twins reared together

Two samples were compared on the CPM. The first sample consisted of 199 pairs of 5- to 7-year-old Canadian twins reared together (MZT/DZT) from the Western Ontario Twin Project, an ongoing longitudinal study initiated in 1987 (Vernon *et al.* 1997). The sample was selected from a larger pool of 3- to 7-year-olds on the assumption that scores below 5 years would be less reliable. Only twins with complete information (e.g. zygosity) were included. One year after the initial testing, 108 participants were tested a second time. (The most recent score was used.) There were 58 MZT pairs (29 female pairs and 29 male pairs) and 141 DZT pairs (31 female pairs, 42 male pairs and 68 opposite-sex pairs), with 148 5-year-olds, 208 6-year-olds and 42 7-year-olds. The second sample consisted of 92 16- to 66-year-old Roma (Gypsies) in Serbia, previously studied by Rushton *et al.* (2007). They were a subset of 323 who had been allotted the CPM after it was found that the SPM produced very low scores for this population.

### (b) Study 2: 152 pairs of twins from the University of Minnesota Study of Twins Reared Apart

Eleven samples were compared on the SPM. The first sample consisted of 152 pairs of adult twins reared apart (MZA/DZA) from the University of Minnesota Study of Twins Reared Apart (MISTRA). This research project was initiated in 1979 and many results reported (Bouchard *et al.* 1990; Segal 2000; Bouchard & McGue 2003). Most of the twins were separated early in life, reared in adoptive families and then reunited only in adulthood. They were assessed with a week-long battery of tests evaluating medical and physical traits as well as psychological characteristics that included GMA, personality, interests and attitudes. The SPM were presented through slides and individually administered on an untimed basis (Lykken 1982). Eight to twelve years after the initial testing, 87 participants returned for a second assessment. The full sample consisted of 385 people (142 males and 243 females), 16- to 77-year-olds (mean=44 years). There were 92 MZ pairs (57 female pairs and 35 male pairs) and 60 DZ pairs (33 female pairs, 12 male pairs and 15 opposite-sex pairs), as well as 33 spouses of twins and 48 other adopted and biological family members. (This was a subset of the fuller MISTRA sample because not all twins completed the SPM.) There were 10 other samples. One of these comprised the 231 16- to 66-year-old Roma in Serbia remaining from study 1; four were from Owen (1992) who administered the SPM to 1093 White, 778 South Asian, 1063 Coloured and 1056 Black 14- to 16-year-old high school students in South Africa; and five were from Rushton *et al.* (2004, 2007) who collected SPM data for undergraduates in South Africa: 11 East Asians, 242 Whites, 99 South Asians, 20 Coloureds and 442 Blacks.

## 3. Results

### (a) Study 1: 199 pairs of 5- to 7-year-old Canadian twins reared together

In the electronic supplementary material, sheet 1 summarizes the results for the twins and the Roma on the 36 puzzles of the CPM. Column A lists the item numbers. Columns B and C give the proportion of the twins and Roma who answered each item correctly. Column D gives the twin–Roma differences in item pass rates (kept positive by subtracting the lower scoring group from the higher). Column E gives the tetrachoric item test–retest correlation, with a minimum score of zero, calculated from the 108 twins tested twice (mean item reliability=0.16). Items relatively difficult or easy for the twins were those found relatively difficult or easy for the Roma (*r*=0.90; *N*=36; *p*<0.001), indicating construct similarity across the two groups. Columns F and G give the item–total correlations, which are the biserial correlations of each item's pass or fail status (0 or 1) with the total score on the test. They indicate the extent to which a particular item measures the same construct measured by the test as a whole, as well as how well the item discriminates between testees within each group. Those with high values among the twins had high values among the Roma (*r*=0.64; *N*=36; *p*<0.001). Columns H and I show the intraclass correlations for the MZT and DZT pairs with a minimum set at zero and the inclusion of opposite-sex pairs in the DZT column. Column J shows the heritabilities calculated by 2×(MZT*r*−DZT*r*), with a minimum score of zero. Column K shows the shared family environmentality measured by MZT*r*-heritability and Column L gives the non-shared environmentalities measured by the differences within twin pairs, i.e. *Σ*|MZT1−MZT2|.

The vectors of both heritability and non-shared environmentality (columns J and L) significantly correlated with the vector of standardized twin–Roma differences (*r*s=0.32; *N*s=36; *p*s<0.05), but the vectors of shared environmentalities did not (*r*=−0.10). Two possible confounding effects were considered: item reliability and the degree of variance in the twins' item pass rates. Given that items with more reliability and more variance enable higher heritabilities and larger group differences to be calculated, a spurious relation *could* be found between vectors of heritability and environmentality on the one hand and of group differences on the other, owing to the relation between both these sets and variance and reliability. To examine this possibility, we used partial correlations to statistically control for item reliability (using the test–retest correlation in column E) and item variance (measured by each item's deviation from the maximally variant pass rate of 50% in column B, i.e. |item pass rate-50|). Partialling out the reliability did not alter the results, whereas partialling out the variation in item pass rate caused the correlation between heritability and group differences to increase (*r*=0.40; *p*<0.01), the correlation with non-shared environmentality to decrease (*r*=0.20; ns) and the correlation with shared environmentality to remain null (*r*=−0.16).

We also examined whether the twin–Roma differences were on *g*, the general factor of mental ability. Since the total score on the Raven's is a good measure of *g*, the item–total correlations (columns F and G) provide an estimate of each item's *g* loading. These item–totals were correlated with the standardized twin–Roma differences, first using the item–totals for the twin group and then those for the Roma. The results were *r*=0.47 (*p*<0.01) and 0.31 (*p*<0.05), respectively, indicating that the twin–Roma differences were on the more *g*-loaded items. (*Note*: it would have been incorrect to use the item–total correlations from the *combined* samples because these would reflect the *between*-groups variance in addition to the *within*-groups variance and thus inflate the effect.)

### (b) Study 2: 152 pairs of twins from the University of Minnesota Study of Twins Reared Apart

In the electronic supplementary material, sheet 2 summarizes the results for the Minnesota twin sample on the 60 puzzles of the SPM. Column A lists the item numbers. Columns B and C give the proportion of the twins who passed each item and the sample size on which it was based. (The first two items were given as practice and not scored.) Column D gives the tetrachoric test–retest correlation for each item, with a minimum score of zero, calculated from data on 87 twins tested twice (mean item reliability=0.40). Column E gives the item–total correlations, which indicate each item's *g* loading, as described in Study 1. Columns F and G give the intraclass correlations for the MZA and DZA twin pairs with a minimum of zero and the inclusion of opposite-sex pairs in the DZ column. Columns H–L provide the four heritabilities and the measure of environmentality (mean item heritability=0.20 and mean item environmentality=0.21). Sheet 3 gives the proportion of each non-twin sample that selected the correct answer on the items. Column A repeats the listing of SPM item numbers. Column B gives the item pass rates for the Roma. Columns C–F show the item pass rates for the South African high school students (White, South Asian, Coloured and Black). Columns G–K show the item pass rates for the South African undergraduates (East Asian, White, South Asian, Coloured and Black). The average item pass rates ranged widely: 93% for East Asian undergraduates, 69% for the twins and 49% for the Roma. Sheet 4 gives the 55 combinations of group comparisons with each group's mean pass rate (column A, with column B giving the mean difference). Columns C–L provide the results of correlating the four heritability vectors and the one of environmentality with those of the group differences in standardized pass rates, along with the levels of significance. (The correlations were kept positive by subtracting the lower scoring group from the higher.)

The vectors of both heritability and environmentality were found to be associated with the magnitude of the group differences (mean *r*s=0.40 and 0.47, respectively; *N*s=58; *p*s<0.05). As in study 1, two possible confounding effects were considered: the item reliabilities (sheet 2, column E) and the degree of variance in the twins' item pass rates (the deviation from the maximally variant pass rate of 50% in column B). The results did not change when item reliability was statistically controlled. However, when controlling for item pass rate variance, the average heritability correlation with the average group difference was reduced to *r*=0.21 (*p*<0.05) and the environmentality correlation was no longer significant (*r*=0.08).

The item pass rates were very similar for all 11 samples (mean *r*=0.87). Those items found relatively difficult (or easy) by one group were found relatively difficult (or easy) by the others, indicating construct validity across the groups. These high correlations occurred despite marked differences in mean levels of passing the items. Moreover, as in study 1, the item–total scores for the twin sample (sheet 2, column E) correlated with the standardized differences in pass rates for all the twin/non-twin comparisons (mean *r*=0.38; *N*=58; *p*<0.05), indicating that the twin/non-twin differences were on *g*, as in study 1. When correlating the 55 group comparisons with the relevant item–total correlations (not shown), the mean *r*=0.36 (total *N* of correlations=110).

## 4. Discussion

We found that vectors of heritability and environmentality calculated from two independent twin samples on tests of GMA were associated with vectors of population group differences on the same tests and, prior to correction, at about the same level of magnitude. The results were robust despite marked heterogeneity in age range across samples, a lack of power due to small *N*s in some groups, and many non-optimal item pass rates. Heritabilities and environmentalities estimated from 5- to 7-year-old twins reared together in Canada generalized to a sample of 16- to 66-year-old Roma in Serbia, while those estimated from 17- to 77-year-old twins reared apart in the Minnesota Study of Twins Reared Apart generalized to another sample of Serbian Roma as well as to high school and university students from South Africa. Thus, these results join other data to suggest that genetic as well as environmental influences contribute to group differences in GMA (Rushton & Jensen 2005; Lynn 2006). They appear to confirm what has long been referred to as the ‘default hypothesis’ by those psychometricians who have studied the issue most intensely (Jensen 1998), i.e. that, by adulthood, genetic and environmental factors carry the same weight in causing population group differences in GMA as they do in causing individual differences (say 50% each).

Item reliabilities and item variance in the twins' pass rates were considered as potential sources of contamination because each could affect item heritability as well as the magnitude of the group differences, thereby producing a spurious relation between them. When item reliability and item variance were statistically controlled, the correlation between heritability and group differences remained intact, although the correlation between environmentality and group differences went to zero. This led one reviewer to suggest that the results could be interpreted in terms of a 100% genetic–0% environmental model. However, in the case of the item reliabilities, there may have been an under-correction as the reliabilities themselves were based on 1-year retests in 5- to 7-year-olds and 10-year retests in adults. In the case of the item variances, there may have been an over-correction and it is always possible that an (unmeasured) methodological factor that affected heritability might also affect the group differences and thus reduce that correlation to zero too.

A range of interpretations concerning the strength of the effects in these data, ranging from ‘weak’ to ‘strong’, is possible. The more stringent conclusion would emphasize that the findings are based only on correlational analyses, which do not prove causality. There may be (unmeasured) gene–environment interactions that can make heritabilities and environmentalities more dependent on each other than is typically assumed (Johnson in press). For example, identical twins reared apart may experience similar environments owing to the similar way they select from the array of possible alternatives, thereby making the phenotypic variance apportioned to heritability partly environmental in origin. Conversely, identical twin differences, apportioned to environmentality, may occur because each twin inherits an equally vulnerable (or resilient) personality and thus suffer a similar level of setback to separate events. However, it is difficult to see how these (unmeasured) potential interactions could explain away our finding that the test items measured the same construct across twins reared together and apart and across very diverse groups, as indicated by their similar levels of item pass rate (*r*=0.87), item–total correlation (*r*=0.39) and item–total association with the magnitude of the group differences (*r*=0.38).

Rough-hewn though our heritability and environmentality estimates may have been, as well as our corrections for item reliability and item variance, the results call into question three widely held assertions that, in various circles, have become dogma: (i) test takers *must* be similar in cultural, educational and social background to those on whom the test was standardized, (ii) heritability estimates are only specific to a population, and (iii) the differences between population groups in GMA are *only* due to ecological factors and *only trivially*, if at all, due to genetic influence.

The results found here are consistent with the preponderance of evidence from other studies on the cross-cultural validity of GMA. Apart from the obvious example of language bias, there is little or no evidence of population-specific cultural effects. For example, Sternberg *et al.* (2001) found that GMA in 12- to 15-year-old Kenyans predicted school grades at about the same level as they do in the West with a mean *r*=0.40 (*p*<0.001). Rushton *et al.* (2004) found that GMA predicted university performance equally well in African and non-African engineering students in South Africa (*r*∼0.30; *p*<0.05). Salgado *et al.* (2003) demonstrated the international generalizability of GMA across 10 member countries of the European Community (EC), thus contradicting the view that criterion-related validity is moderated by differences in a nation's culture, religion, language, socioeconomic level or employment legislation. He found that scores predicted job performance ratings at *r*=0.62 and training success at *r*=0.54.

Twin designs are an underused resource in the human sciences (Segal 2000; Bouchard & McGue 2003). The present study demonstrates their usefulness in showing that a similar transaction of genetic and non-genetic influence applies across a wide range of population groups growing up in diverse cultures. There appears to be a set of human psychological adaptations underlying the cognitive problem solving required for the type of GMA test used here, with individual and group differences comprising normal variants.

## Supplementary Material

Twin Data Spreadsheet
